# Primary or secondary chronic functional dizziness: does it make a difference? A DizzyReg study in 356 patients

**DOI:** 10.1007/s00415-020-10150-9

**Published:** 2020-08-27

**Authors:** Maximilian Habs, Ralf Strobl, Eva Grill, Marianne Dieterich, Sandra Becker-Bense

**Affiliations:** 1 German Center for Vertigo and Balance Disorders, Ludwig-Maximilians-Universität München, Munich, Germany; 2 Department of Neurology, Ludwig-Maximilians-Universität München, Marchioninistrasse 15, 81377 Munich, Germany; 3 Munich Cluster for Systems Neurology (SyNergy), Munich, Germany

**Keywords:** Functional dizziness, Vestibular syndromes, Quality of life, Dizziness handicap inventory, Epidemiology, Age, Gender

## Abstract

In 2017, the term “persistent postural-perceptual dizziness” (PPPD) was coined by the Bárány Society, which provided explicit criteria for diagnosis of functional vertigo and dizziness disorders. PPPD can originate secondarily after an organic disorder (s-PPPD) or primarily on its own, in the absence of somatic triggers (p-PPPD). The aim of this database-driven study in 356 patients from a tertiary vertigo center was to describe typical demographic and clinical features in p-PPPD and s-PPPD patients. Patients underwent detailed vestibular testing with neurological and neuro-orthoptic examinations, video-oculography during water caloric stimulation, video head-impulse test, assessment of the subjective visual vertical, and static posturography. All patients answered standardized questionnaires (Dizziness Handicap Inventory, DHI; Vestibular Activities and Participation, VAP; and Euro-Qol-5D-3L). One hundred and ninety-five patients (55%) were categorized as p-PPPD and 162 (45%) as s-PPPD, with female gender slightly predominating (♀:♂ = 56%:44%), particularly in the s-PPPD subgroup (64%). The most common somatic triggers for s-PPPD were benign paroxysmal positional vertigo (27%), and vestibular migraine (24%). Overall, p-PPPD patients were younger than s-PPPD patients (44 vs. 48 years) and showed a bimodal age distribution with an additional early peak in young adults (about 30 years of age) beside a common peak at the age of 50–55. The most sensitive diagnostic tool was posturography, revealing a phobic sway pattern in 50% of cases. s-PPPD patients showed higher handicap and functional impairment in DHI (47 vs. 42) and VAP (9.7 vs. 8.9). There was no difference between both groups in EQ-5D-3L. In p-PPPD, anxiety (20% vs. 10%) and depressive disorders (25% vs. 9%) were more frequent. This retrospective study in a large cohort showed relevant differences between p- and s-PPPD patients in terms of demographic and clinical features, thereby underlining the need for careful syndrome subdivision for further prospective studies.

## Introduction

Functional (somatoform) dizziness is a frequent cause of chronic, ongoing dizziness seen in specialized dizziness units. It considerably burdens patients in the absence of an acute peripheral or central vestibular pathology [1, 2]. The clinical terminology for functional dizziness changed frequently in the past and was comprised of heterogeneous terms. The Bárány Society redefined chronic functional dizziness under the new name of persistent postural-perceptual dizziness (PPPD) in 2017 ([3], Table 1). This new definition is based on the long-standing expert knowledge of several forms of chronic functional dizziness, such as phobic postural vertigo [4–6], visual vertigo [7], chronic subjective dizziness [8–10], and space-motion discomfort [11]. It comprises the essence of each entity mentioned above with long-lasting non-rotatory dizziness, unsteadiness, and fluctuations of symptoms with stimulus-triggered provocation and/or exacerbation (e.g., visual stimuli). Common precipitating conditions for PPPD are earlier vestibular experiences, initiated especially by organic peripheral or central vestibular disorders or psychological distress [12–15]. On the other hand, patients with psychiatric comorbidities, predominantly depressive or anxiety disorders, are also known to have a higher risk of developing functional dizziness [10, 16, 17].

**Table 1:** Current diagnostic criteria of the Bárány Society for persistent postural-perceptual dizziness (PPPD)

(a) One or more symptoms of dizziness, unsteadiness, or non-spinning vertigo on most days for at least 3 months Symptoms last for prolonged (hours long) periods of time, but may wax and wane in severity Symptoms need not be present continuously throughout the entire day
(b) Persistent symptoms occur without specific provocation, but are exacerbated by three factors: Upright posture Active or passive motion without regard to direction or position Exposure to moving visual stimuli or complex visual patterns
(c) The disorder is precipitated by conditions that cause vertigo, unsteadiness, dizziness, or problems with balance including acute, episodic, or chronic vestibular syndromes, other neurologic or medical illnesses, or psychological distress When the precipitant is an acute or episodic condition, symptoms settle into the pattern of criterion as the precipitant resolves, but they may occur intermittently at first, and then consolidate into a persistent course When the precipitant is a chronic syndrome, symptoms may develop slowly at first and worsen gradually
(d) Symptoms cause significant distress or functional impairment
(e) Symptoms are not better accounted for by another disease or disorder

All five criteria (a–e) must be fulfilled to make the diagnosis of PPPD

However, little is known about the clinical characteristics of chronic functional dizziness syndromes resulting from preceding somatic (vestibular) disorders (i.e., secondary, s-PPPD; e.g., Huppert et al. [6]), and those originating on their own as the primary cause of the illness (i.e., primary, in absence of a preceding somatic trigger, p-PPPD). Although at first glance the clinical presentation of p- and s-PPPD appears very similar, clinical experience suggests variable accentuation in terms of functionality, handicap, psychiatric comorbidities, and introspection in relation to the underlying cause [14–16, 18]. Therefore, with this study in a large, well-investigated PPPD cohort, we aimed to elucidate typical demographical and clinical features, as well as potential differences between primary and secondary variants of functional dizziness.

## Methods

### Study design

Using the DizzyReg database [19, 20], we screened 470 patients with chronic functional dizziness diagnosed at a tertiary dizziness center (German Center for Vertigo and Balance Disorders, DSGZ, Munich, Germany) over 24 months between 2015 and 2017. Inclusion criteria were a newly diagnosed or known form of chronic functional dizziness, disease duration of at least 3 months, age above 18 years, and written informed consent. Additional exclusion criteria for participation were as follows: acute unilateral or chronic bilateral vestibulopathy (as assessed by caloric testing, video head-impulse test, and clinical examination), relevant neurological and neurodegenerative diseases (e.g., encephalomyelitis or severe polyneuropathy).

In retrospect, 356 patients fulfilled the current diagnostic criteria for PPPD ([3], Table 1) and were included in the statistics. All patients underwent a psychiatric investigation, as well as a detailed neurological and neuro-orthoptic examination including fundus photography by a scanning laser ophthalmoscope (SLO) for the measurement of ocular torsion and electronic assessment of the subjective visual vertical (SVV). Furthermore, all patients underwent either video-oculography at rest to determine spontaneous nystagmus and during water caloric stimulation (VOG-CS), video head-impulse test (vHIT), or both for evaluation of peripheral vestibular function, as well as static posturography. These detailed examinations were especially useful to exclude acute vestibular tone imbalance, e.g., ocular tilt reaction. Assisted by a neurologist, all patients answered a short questionnaire for vestibular disorders regarding vertigo and dizziness phenomenology and associated symptoms. All patients also completed the Dizziness Handicap Inventory (DHI), Vestibular Activities and Participation (VAP), and the Euro-Qol-5D-3L questionnaire (EQ-5D-3L).

Depending on the information about an ascertained preceding somatic/vestibular trigger (e.g., benign paroxysmal positional vertigo) provided by the patients themselves or available from medical documentation, each patient was classified as either s- or p-PPPD.

### Protocol approval and patient consent

The study was approved by the appropriate ethics committee, and has, therefore, been performed in accordance with the ethical standards laid down in the 1964 Declaration of Helsinki and its later amendments. The DizzyReg database prospectively collects all information from the clinical patient records or from discharge letters, subject to informed consent from the patient.

### Instrument-based vestibular testing

Caloric testing for the function of the horizontal semicircular canals was done using 30 °C cool and 44 °C warm water irrigation measuring peak slow-phase velocity (SPV) by video oculography (EyeSeeCam®). Values less than 5°/s SPV were considered pathological. Side asymmetry during caloric testing was assessed by the vestibular paresis formula of Jongkees [21]. Values above 30% were considered pathological. Subjective visual vertical (SVV), a test for an acute dysfunction of graviceptive pathways, was measured in a motor-operated hemispheric dome as deviation from the objective vertical axis in degrees. A mean deviation of more than ± 2.5° from the true vertical was considered a pathological SVV deviation.

Standardized vHIT measurements of the semicircular function in the high-frequency range were obtained in a bright room with a red target affixed at eye level at a distance of 1.8 m using the EyeSeeCamHIT® system (Interacoustics, Middelfart, Denmark) [22], with the procedure as described in [23]. A vHIT gain of less than 0.7 was considered pathological. The vHIT gain asymmetry index was calculated as follows: (difference of ipsilateral and contralateral gain/sum of ipsilateral and contralateral gain) × 100%. A vHIT gain asymmetry index of more than 10% was considered pathological.

Posturographic measurements were performed using a stabilometer platform (Kistler 9261A; Kistler Group, Winterthur, Switzerland) in an upright standing position. Displacement of center of gravity was assessed by the total sway path for *x*-, *y*-, and *z*-directions (for *x*- and *y*-axis: m/min and for *z*-axis: kN/min). Balance control was evaluated using a well-established posturography protocol comprising ten different conditions with increasing difficulty. In addition to the regular analysis, sway patterns were analyzed over all conditions by an artificial neuronal network [24, 25] and categorized as normal, phobic, cerebellar, orthostatic, or vestibular patterns. The “phobic pattern” is typically characterized by a poor performance (i.e., increased body sway) under easy conditions and almost normal performance at higher difficulty levels or with an additional dual task [26].

### Standardized Questionnaires

The DHI is a well-established measure for self-perceived limitations posed by vertigo and dizziness [27]. A total of 25 questions are used to evaluate different aspects of disability. The range of the DHI is from 0 to 100, with a score of 0 being the best possible score (0 = no, 2 = sometimes, 4 = yes). It is divided into 3 subcategories: emotional (36 points), functional (36 points), and physical handicap (28 points). The higher the score, the greater the problems.

Functioning and participation were assessed with the VAP short form. The VAP is specifically designed for patients with vertigo and dizziness und is validated for different countries and languages [28, 29]. It measures functioning and participation in two separate subscales, each consisting of 6 items (ordinal Likert scale 0–4). Using weights derived from Rasch analysis, the first scale has a range of 0–23 points and the second of 0–20 points, with higher scores indicating more restrictions.

Health-related quality of life was assessed with the generic EuroQoL five-dimensional questionnaire (EQ-5D-3L) [30]. This is subdivided into five health state dimensions, namely mobility, self-care, usual activities, pain/discomfort and anxiety/depression, with each dimension having three levels: no problems, some problems, and extreme problems. These health states were converted into EQ5D scores using the German time trade-off scoring algorithm. The resulting total score ranges from 0 to 1, with higher scores indicating better quality of life. The EQ-5D-3L visual analog scale (VAS) was documented in a score of 0–100, with a score of 100 reflecting the best self-reported health condition.

### Statistical analysis

All continuous variables are reported with mean values and standard deviation and categorical variables with absolute and relative frequencies. The median was used for ordinal variables. All variables were checked for normal distribution (Kolmogorov–Smirnov test) and equality of variances. Group comparison of parametric variables (differences of mean) was assessed by the student’s *t* test. In case of non-normality, differences were evaluated by the Mann–Whitney *U* test. For differences of nominal variables between two groups (2 × 2 contingency tables), we performed Fisher’s exact test. When comparing more than two groups or factors, a chi-squared test was used. Correlations were measured with Pearson’s *r*. For all statistical tests, a two-sided p value below 0.05 was considered significant. All statistical calculations were performed with the Statistical Package for the Social Sciences (SPSS®), version 25 (IBM Corporation) and with R 3.6.1. (R Core Team 2019; https://www.r-project.org/index.html).

## Results

**Fig. 1:**
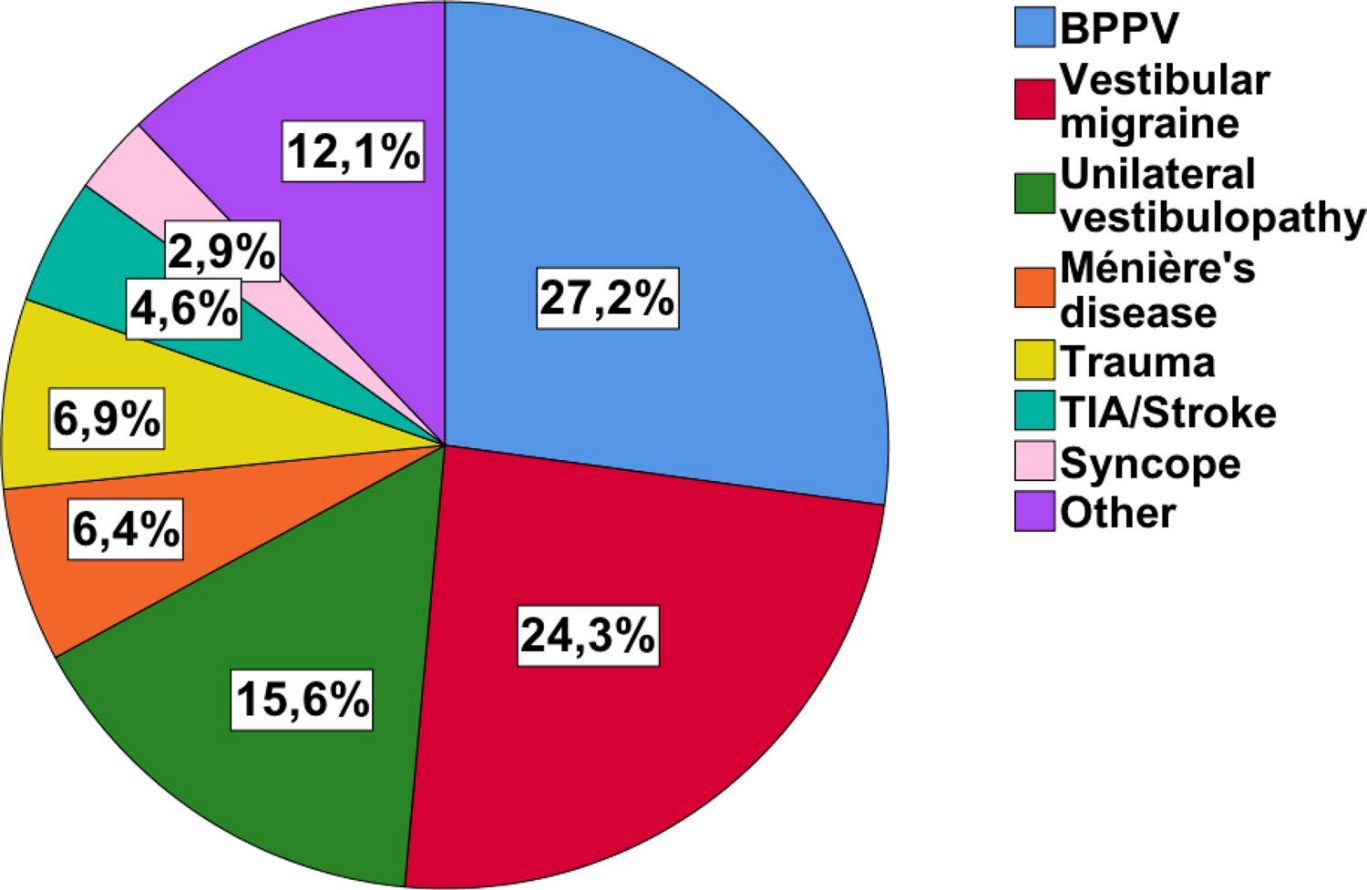
Somatic triggers for secondary functional dizziness. Pie chart of somatic triggers for secondary PPPD (*n* = 162) reported as relative percentages. Benign paroxysmal positional vertigo (BPPV), vestibular migraine, and acute unilateral vestibulopathy were the most common preceding organic diseases. Examples of relatively rare other organic triggers were mal de debarquement syndrome, vestibular paroxysmia, or superior canal dehiscence syndrome. *TIA* transient ischemic attack

Out of 356 PPPD patients, 195 patients (55%) were categorized as p-PPPD and 162 (45%) as s-PPPD. Figure 1 summarizes all somatic triggers of vertigo in s-PPPD patients.

### All PPPD patients

For the whole patient cohort, the mean disease duration was 2.8 years before admission. The mean age was 46 years (range 18–81 ± 14 years), slightly favoring females (56%). The predominant vestibular sensation was lightheadedness (75%), followed by unsteadiness (72%). 49% reported additional subjective rotational vertigo sensations. The most common symptom-length of a vertigo episode was > 48 h (33%), followed by 1–12 h (28%). The vertigo/dizziness episodes were often accompanied by other symptoms, e.g., headache (50%) or head pressure (46%), phono- or photophobia (39%), tinnitus (40%), or nausea (27%). Many patients reported experiences of motion sickness (59%), memory and concentration deficits (50%), as well as susceptibility to visual triggers (28%). The most common visual triggers were quick movements of surroundings (36%), crowds (24%), design of buildings (22%), and flashes of light (17%). About half of all patients had mild, but regular alcohol consumption (51%). Interviews at admission and documentation revealed an overall high prevalence of psychiatric comorbidities with depressive episodes (18%) and anxiety disorders (15%) in the past or present, but only 31 patients (9%) were currently on antidepressant medication. For more details, see Table 2.

**Table 2:** Demographics and patient characteristics

	All patients (*n* = 356)	Primary PPPD (*n* = 194)	Secondary PPPD (*n* = 162)	*p* value
Age in years (mean, SD)	45.8 ± 14.2	44.2 ± 14.6	47.7 ± 13.6	**0.022**
Gender (% female)	55.9	49.0	64.2	**0.005**
Disease duration in years (mean, SD)	2.8 ± 3.3	2.8 ± 3.3	2.8 ± 3.3	0.924
Psychiatric comorbidities				
Depressive disorder (%)	17.7	24.7	9.3	**< 0.001**
Anxiety disorder (%)	15.4	20.1	9.9	**0.008**
Regular alcohol consumption (%)	51.3	56.0	45.6	0.055
Type of vertigo				
Rotational (%)	48.6	33.5	66.7	**< 0.001**
Unsteadiness (%)	71.6	76.3	66.0	**0.034**
Lightheadedness (%)	74.2	75.8	72.2	0.467
Length of vertigo episodes				
< 2 min (%)	16.0	14.9	17.3	0.565
2–20 min (%)	17.4	15.5	19.8	0.327
20–60 min (%)	9.8	8.2	11.7	0.288
1–12 h (%)	28.1	30.9	24.7	0.236
12–48 h (%)	6.5	8.8	3.7	0.081
> 48 h (%)	33.4	34.6	32.5	0.735
Sensitivity to passive motion (%)	59.0	55.7	63.0	0.194
Susceptibility to visual triggers (%)	27.8	35.1	19.1	**0.001**
History of falls (last 12 months, %)	22.4	18.8	26.7	0.095
Accompanying symptoms				
Headache (%)	49.7	45.9	54.3	0.136
Head pressure (%)	45.8	44.8	46.9	0.749
Nausea (%)	26.7	23.2	30.9	0.118
Phono-/photophobia (%)	39.3	36.6	42.6	0.276
Dysesthesia (%)	28.7	32.5	24.1	0.099
Transient loss of hearing (%)	14.0	9.8	19.1	**0.014**
Tinnitus (%)	39.6	38.1	41.4	0.587
Memory and/or concentration deficits (%)	49.7	51.0	48.1	0.596

Demographics and patient characteristics for the whole patient group as well as for the primary and secondary PPPD subgroups separately. Significant differences between p- and s-PPPD are printed in bold

*SD*  standard deviation

Vestibular testing revealed that no PPPD patient showed signs of acute vestibular tone imbalance (i.e., no spontaneous nystagmus in VOG or SLO, no nystagmus provoked by head-shaking, no ocular tilt reaction). SVV measurements were normal in the great majority of patients with an overall mean SVV deviation of 1.3° ± 1.07°. Forty patients (13%) showed borderline mean values slightly above 2.5° (3.37° ± 0.82°; range 2.53°–5.31°), but with great intra-individual variance in between the adjustments not providing evidence of a clinically relevant vestibular imbalance. 70% of all patients tolerated warm and cold caloric semicircular canal testing with reliable results. Caloric testing was abnormal in only 31 out of the 249 patients (12%) due to older residual unilateral peripheral lesions resulting in an overall regular mean side asymmetry index of 16% and mean SPV of the induced nystagmus of 13°/s. vHIT was performed in 90% of all patients and showed comparable results with an abnormal asymmetry index in 39 out of 320 patients (12%). Mean vHIT gain at 60 ms was normal on both sides (0.97 left and 0.91 right side) and the mean vHIT asymmetry index was also normal (4.8%). 70% of all PPPD patients completed posturography sufficiently, of which 49% were normal, 50% showed a typical phobic/functional, and only 1% a vestibular sway pattern. Posturography sway path correlated moderately with patient age for all sway directions (*x*-, *y*- and *z*-axis) with *r* = 0.320 (*p* < 0.001) and showed only inconsistent or weak correlations with standardized questionnaires (e.g., sway path y-axis and DHI total score with *r* = 0.207; *p* = 0.002). For more details, see Table 3.

**Table 3:** Instrument-based vestibular diagnostics

	All patients (*n* = 356)	Primary PPPD (*n* = 194)	Secondary PPPD (*n* = 162)	*p* value
Caloric stimulation (mean SPV, SD, °/sec.)	12.7 ± 6.7	14.4 ± 7.1	11.1 ± 5.9	**0.004**
Caloric stimulation (mean side asymmetry index, SD)	15.6 ± 11.5	13.6 ± 9.3	18.0 ± 13.5	**0.004**
Abnormal caloric testing (%)	12.4	6.1	21.3	**< 0.001**
SVV deviation (mean in°, SD)	1.32 ± 1.07	1.32 ± 1.05	1.32 ± 1.10	0.986
vHIT gain 60 ms left (mean ratio, SD)	0.97 ± 0.13	0.98 ± 0.13	0.95 ± 0.13	**0.018**
vHIT gain 60 ms right (mean ratio, SD)	0.91 ± 0.16	0.92 ± 0.15	0.89 ± 0.16	**0.031**
vHIT (mean side asymmetry index, SD)	4.78 ± 4.49	4.66 ± 4.43	4.92 ± 4.59	0.603
Abnormal vHIT (%)	12.2	7.6	18.2	**0.006**
Posturography firm ground, eyes open (EO)				
Sway path *x*-direction (m/min)	0.69 ± 0.40	0.69 ± 0.33	0.70 ± 0.48	0.908
Sway path *y*-direction (m/min)	0.84 ± 0.34	0.88 ± 0.38	0.80 ± 0.27	0.064
Sway path *z*-direction (kN/min)	0.23 ± 0.09	0.23 ± 0.08	0.22 ± 0.09	0.861
Posturography firm ground, eyes closed (EC)				
Sway path *x*-direction (m/min)	0.72 ± 0.42	0.73 ± 0.39	0.70 ± 0.46	0.538
Sway path *y*-direction (m/min)	1.11 ± 0.62	1.12 ± 0.64	1.11 ± 0.60	0.704
Sway path *z*-direction (kN/min)	0.23 ± 0.14	0.23 ± 0.13	0.23 ± 0.14	0.95
Posturography foam rubber, eyes open (FEO)				
Sway path *x*-direction (m/min)	1.05 ± 0.53	1.05 ± 0.47	1.05 ± 0.59	0.916
Sway path *y*-direction (m/min)	1.42 ± 0.86	1.45 ± 0.96	1.38 ± 0.72	0.558
Sway path *z*-direction (kN/min)	0.28 ± 0.27	0.30 ± 0.34	0.26 ± 0.13	0.211
Posturography foam rubber, eyes closed (FEC)				
Sway path *x*-direction (m/min)	1.66 ± 0.89	1.67 ± 0.84	1.66 ± 0.95	0.982
Sway path *y*-direction (m/min)	2.36 ± 1.32	2.42 ± 1.46	2.23 ± 1.11	0.472
Sway path *z*-direction (kN/min)	0.42 ± 0.35	0.44 ± 0.42	0.39 ± 0.26	0.25
Posturography phobic pattern (%)	49.8	49.7	50.0	1.000

Results of instrument-based vestibular testing for the whole patient group, as well as for primary and secondary PPPD subgroups separately. Significant differences between p- and s-PPPD are printed in bold

*SPV* peak slow-phase velocity, *SD* standard deviation, *SVV* subjective visual vertical, *vHIT* video head-impulse test

All patients answered the standardized questionnaires EQ-5D-3L, VAP short form, and DHI (Table 4). The mean VAS score of the EQ-5D-3L was reduced to 58 ± 20 points (age- and country-related population norm 78 points), and the mean EQ-5D-3L index was reduced to 0.80 ± 0.22 (age- and country-related population norm; 0.90) [30]. The VAP mean ordinal subscale 1 reflecting functioning was 9.3 ± 3.1 points and the mean ordinal subscale 2 reflecting participation was 8.2 ± 3.2 points, indicating a considerable overall impairment. The highest handicap was scored in the VAP categories “focusing attention” (1.81 ± 1.11), “walking long distances” (1.47 ± 1.28), and “bending over” (1.47 ± 1.11). The DHI scores were all elevated: total score 44.5/100 (± 18.9), emotional subscore 16.2/36 (± 7.5), functional subscore 17.4/36 (± 8.5), and physical subscore 11.3/28 (± 6.5).

**Table 4:** Standardized questionnaires

	All patients (*n* = 356)	Primary PPPD (*n* = 194)	Secondary PPPD (*n* = 162)	*p* value
DHI (mean, SD)				
Total score	44.5 ± 18.9	42.1 ± 19.3	47.3 ± 18.3	**0.014**
Emotional subscore	16.2 ± 7.5	16.2 ± 7.9	16.2 ± 7.1	0.927
Functional subscore	17.4 ± 8.5	16.3 ± 8.5	18.8 ± 8.4	**0.008**
Physical subscore	11.3 ± 6.5	10.1 ± 6.4	12.7 ± 6.5	**< 0.001**
VAP short version (mean, SD)				
Subscale 1 total	9.3 ± 3.1	8.9 ± 3.1	9.7 ± 3.1	**0.038**
1 Focusing attention	1.81 ± 1.11	1.78 ± 1.08	1.85 ± 1.14	0.570
2 Lying down	1.00 ± 1.10	0.79 ± 0.97	1.24 ± 1.18	** < 0.001**
3 Standing up	1.15 ± 1.05	1.14 ± 1.04	1.16 ± 1.07	0.863
4 Bending over	1.47 ± 1.11	1.29 ± 1.08	1.68 ± 1.12	**0.001**
5 Lifting objects	1.07 ± 1.06	0.99 ± 1.12	1.16 ± 1.08	0.130
6 Sports	1.61 ± 1.04	1.58 ± 1.03	1.66 ± 1.05	0.454
Subscale 2 total	8.2 ± 3.2	8.2 ± 3.2	8.2 ± 3.2	0.981
7 Walking long distances	1.47 ± 1.28	1.49 ± 1.37	1.44 ± 1.17	0.703
8 Climbing stairs	1.38 ± 0.86	1.32 ± 0.89	1.46 ± 0.82	0.143
9 Running	1.00 ± 0.61	0.90 ± 0.64	1.03 ± 0.56	0.062
10 Moving around	1.03 ± 1.07	1.03 ± 1.10	1.03 ± 1.03	0.992
11 Traveling as passenger	1.35 ± 1.18	1.33 ± 1.22	1.38 ± 1.14	0.647
12 Driving a car or riding a bike	1.38 ± 1.02	1.40 ± 1.01	1.36 ± 1.04	0.769
EQ5D-3L				
QoL VAS (mean percentage, SD)	57.7 ± 19.7	58.2 ± 19.4	57.2 ± 20.1	0.651
QoL summary index (mean, SD)	0.80 ± 0.22	0.79 ± 0.24	0.82 ± 0.20	0.126

Results of the dizziness handicap inventory (DHI), the Vestibular Activities and Participation questionnaire (VAP), and the Euro-Qol-5D-3L questionnaire (EQ-5D-3L) for the whole patient group as well as for the primary and secondary PPPD subgroups separately. Significant differences between p- and s-PPPD are printed in bold

*QoL*  quality of life, *VAS* visual analog scale, *SD* standard deviation

### Comparative analyses in primary and secondary PPPD patients

**Fig. 2:**
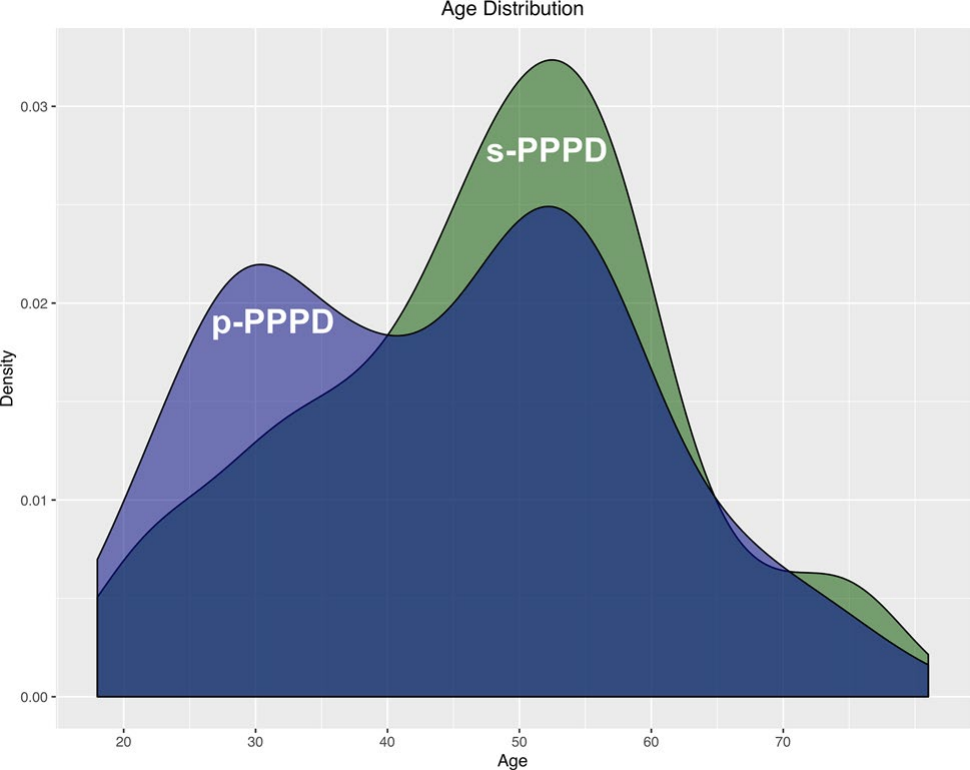
Age distribution density curve in primary and secondary PPPD. Primary (blue) and secondary PPPD (green) show a common peak at 50–55 years of age, whereas p-PPPD shows an additional peak in in young adults between 25 and around 30 years of age

Although both types of PPPD occurred at any age, there were significant differences in age distribution (Kolmogorov–Smirnov test; *p* = 0.037) (Table 2): the mean age in p-PPPD was 44 years (range 18–81 years) vs. 48 years (range 19–77 years) in s-PPPD (Student’s *t* test: *p* = 0.022). Even though both patient groups showed a corresponding frequency peak at 50–55 years of age, in p-PPPD, there was an additional bimodal peak in the age curve at 25–30 years (Fig. 2). s-PPPD patients were more often female (64% vs. 49%; *p* = 0.005), and showed a higher rate of additional rotational vertigo sensations (67% vs. 34%; *p* < 0.001). There was no difference regarding the length of vertigo episodes. p-PPPD patients were more prone to visual stimuli as vertigo triggers (35% vs. 19%; *p* = 0.001), and showed a trend towards a higher frequency of regular alcohol consumption (56% vs. 46%; *p* = 0.055). Psychiatric comorbidities were more common in p-PPPD than in s-PPPD patients in terms of depressive (25% vs. 9%; *p* < 0.001), and anxiety disorders (20% vs. 10%; *p* = 0.008).

Due to the preceding organic disease, vestibular testing remained abnormal significantly more often in s-PPPD patients (e.g., mean SPV of caloric nystagmus, mean vHIT gain, as well as asymmetry indices of caloric stimulation and vHIT) (Table 3). There was no difference regarding mean SVV deviation; also, borderline SVV adjustments with great intra-individual variance were equally distributed. In both patient groups, posturography revealed a phobic pattern in about 50%.

**Fig. 3:**
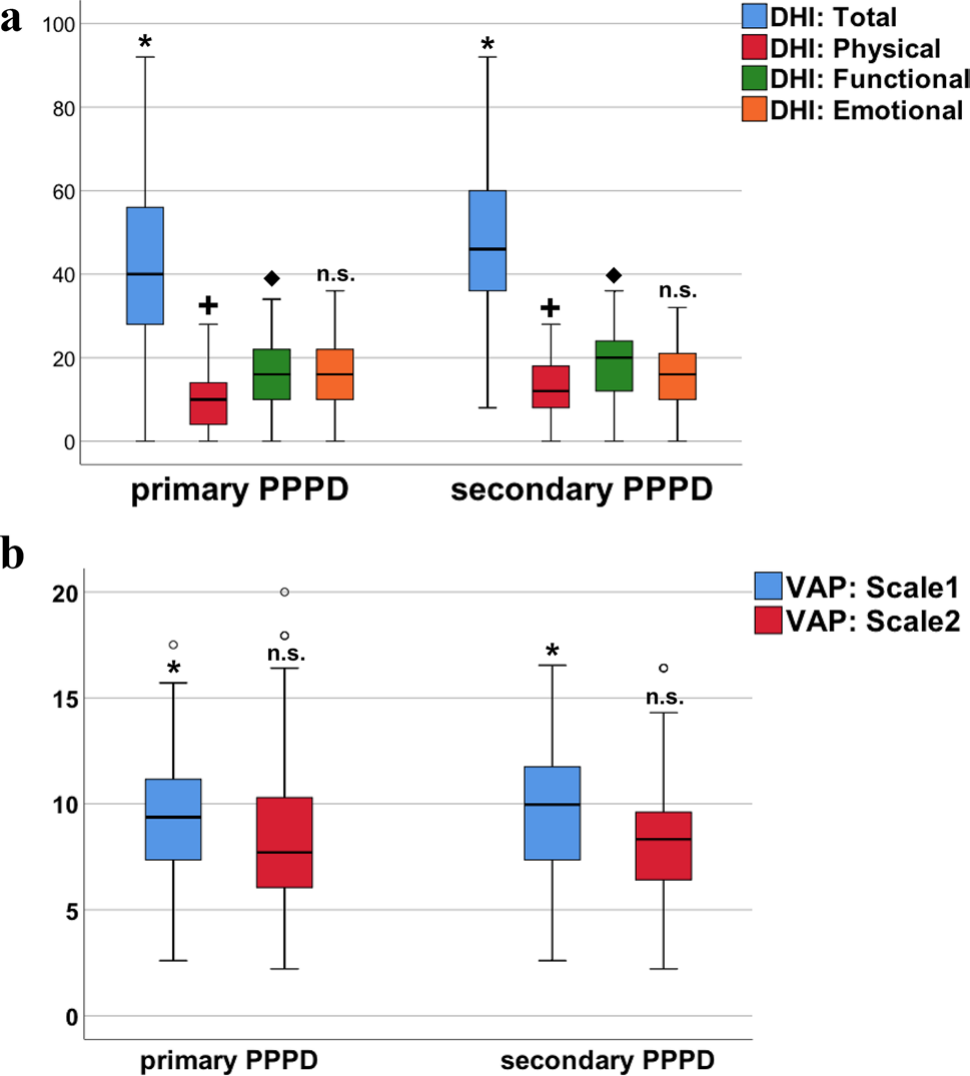
Boxplots of handicap, functioning, and participation in primary and secondary PPPD. The **a** Dizziness Handicap Inventory **(**DHI) total score and subscores (emotional, functional, physical), as well as **b** Vestibular Activities and Participation (VAP) subscales (1 = functioning and 2 = participation) in primary and secondary PPPD, both showing significantly higher impairment in s-PPPD than in p-PPPD in terms of the DHI total score (*p = 0.014), the DHI physical (^+^*p* < 0.001) and functional subscores (*p* = 0.008), as well as the VAP subscale 1 (*p = 0.038). *n.s.* not significant. Whiskers indicate 95% confidence intervals

Both handicap questionnaires for vestibular functioning (DHI and VAP subscale 1, Fig. 3) showed significantly higher scores in s-PPPD patients (DHI total: 47.3 vs. 42.1; *p* = 0.014; VAP: 9.7 vs. 8.9; *p* = 0.038). Especially the physical (e.g., quick movements, turning around) and functional (e.g. working, social life) subscores of the DHI were remarkably increased in s-PPPD compared to p-PPPD with 12.7 vs. 10.1 (< 0.001) and 18.8 vs. 16.3 (*p* = 0.008), respectively. There was no significant difference between p- and s-PPPD regarding quality of life, as measured by EQ5D-3L VAS or the EQ5D-3L index.

## Discussion

The key findings of our monocentric, database-driven, retrospective study in 356 PPPD patients concerning typical demographic characteristics, clinical features, and vestibular diagnostics with regard to primary (p-PPPD) or secondary (s-PPPD) functional dizziness development were as follows:

1. The mean age of p-PPPD patients was significantly lower than that of s-PPPD patients (44.4 vs. 47.7 years). Besides a common age peak in both subgroups in older adults of 50–55 years of age, p-PPPD showed an additional peak in age distribution in young adults between 25 and 30 years (bimodal peak) (Fig. 2).
2. PPPD generally preferred female gender (♀:♂ = 56%:44%), especially in the s-PPPD subgroup (64%).
3. In about half of all s-PPPD cases, preceding BPPV (27%) and vestibular migraine (24%) were the triggering diseases, followed by acute unilateral vestibulopathy in about 16% (Fig. 1).
4. p-PPPD patients showed psychiatric comorbidities significantly more often than s-PPPD patients, e.g., anxiety (20% vs. 10%) and depressive disorders (25% vs. 9%).
5. s-PPPD were more handicapped and functionally impaired in daily activities as assessed by DHI and VAP (Fig. 3). However, quality of life was equally reduced in both patient groups.
6. A surprisingly high number of PPPD patients scored additional subjective rotational vertigo sensations beside the ongoing symptoms, not only in the s-PPPD, but also in the p-PPPD subgroup (67% vs. 34%).
7. In general, p-PPPD patients reported significantly higher susceptibility to visual triggers (e.g., crowds and quick movements of surroundings; 35% vs. 19%).

Our PPPD cohort showed a higher prevalence of p-PPPD (55% vs. 45%). Thus, even though a psychiatric exploration, careful history-taking, and a vestibular examination routinely took place in our patients, a preceding organic vestibular disease potentially triggering the secondary psychosomatic disease could only be found in the minority of patients. Comparable reports on frequency distribution are rare. The only available study by Staab and Ruckenstein from 2007 [10] in 345 patients with chronic subjective dizziness of uncertain cause, also seen in a tertiary balance center, reported in about one third of patients no neurootologic or other medical condition explaining the symptoms (e.g., potentially comparable to our p-PPPD subgroup), whereas in the majority of cases (approximately two thirds) mainly central or peripheral vestibular deficits, migraine, traumatic brain injury, or dysautonomia/dysrhythmia were evident. The shift towards a higher portion of p-PPPD cases in our study might be best explained by variant patient inclusion criteria (patients with currently relevant vestibular dysfunction, e.g., due to unilateral vestibulopathy with acute vestibular tone imbalance or chronic bilateral vestibular failure, were excluded), and referral conditions (e.g., referral to our center only by a resident medical specialist, ENT doctor, or neurologist), as well as by changes in diagnostic criteria for organic diseases over time.

The mean age of all PPPD patients is in line with previous case series in functional dizziness [5, 10]. The older mean age in s-PPPD can be best explained by the prerequisite of a preceding organic disease, which often favors older ages, e.g., BPPV [31]. Although p- and s-PPPD can occur at any ages, both groups showed a peak of the age distribution curve at 50–55 years. Interestingly, p-PPPD patients had an additional early peak around the age of 30 years, thereby potentially indicating different mechanisms generating chronic functional vertigo. The age divergence between s- and p-PPPD fits nicely with two psychosomatic follow-up studies in smaller patient cohorts that reported an average age of 52 years in s-PPPD [32] and 42 years in p-PPPD [33]. Furthermore, incidences of somatoform and anxiety disorders are reported to often favor younger age, with a pronounced onset age peaking in 20–40 year-olds [34, 35].

In fact, our p-PPPD patients showed psychiatric comorbidities, e.g., anxiety and depressive disorders, significantly more often than the s-PPPD patients (20% vs. 10%; 25% vs. 9%). However, the overall percentage of psychiatric comorbidities in our cohort was relatively low compared to studies applying detailed psychiatric test procedures (e.g., structured clinical interviews, SCID), and thus reporting prevalence rates of up to 50–60% [10, 36]. In our study, psychiatric comorbidity rates were based on written or verbal information provided by the patients themselves, or were newly diagnosed by experienced neurologists when the patients visited our center, and were in line with earlier data showing a low agreement between the diagnosis of neurologists and SCID interviews for psychiatric disorders [37]. However, one may speculate that hidden psychiatric comorbidities among our PPPD cohort might potentially influence the relation between p- and s-PPPD. Nevertheless, our results emphasize the importance of clinical screening for psychiatric comorbidities in PPPD patients in general, and especially in younger adults without central or peripheral vestibulopathies.

Overall, female sex was slightly predominant in this PPPD cohort, especially in s-PPPD patients. This effect might be partly explained by female preponderance in some organic vertigo disorders, such as vestibular migraine [38, 39] or BPPV [31, 38, 39]. However, earlier studies not only in functional dizziness [5, 10], but also in other somatoform diseases with neurological symptoms, e.g., somatoform pain disorder [40] or non-epileptic seizures [41], also report a concurrent predominance of female gender, the reason for which might be complex.

Dizziness experience in p-PPPD and s-PPPD appeared very similar in terms of disease duration, length of episodes, history of falls, and overall accompanying symptoms. However, a strikingly high number of patients not only in the s-PPPD group (67%, most likely due to organic rotational vertigo attacks induced by MD, VM or BPPV), but also in the p-PPPD group (34%) reported additional rotational vertigo sensations. This appears clinically important and underlines the fact that the subjective clinical sensations described by laypersons with functional dizziness might be more varied than actually reflected by the diagnostic criteria requiring prolonged dizziness, unsteadiness, or non-spinning vertigo. Clinicians should therefore be very careful with chief classification on the basis of the patients’ description of their symptoms and should search for psychiatric comorbidity, especially in those reporting rotational vertigo, but not fulfilling the diagnostic criteria for an organic vertigo syndrome. The traditional rule that rotational vertigo does not occur without spontaneous or gaze-evoked nystagmus at the time it is experienced could help in finding the correct diagnosis [42].

Furthermore, p-PPPD showed significantly more susceptibility to visual triggers (e.g., crowds and quick movements of surroundings) than s-PPPD, most probably due to a more focused self-perception with increased sensitivity regarding visual inputs. s-PPPD reported an objectively higher handicap in daily activities (DHI and VAP score), particularly in terms of physical und functional domains, due to their pre-existing or ongoing vestibular disorders (e.g., vertigo attacks by VM or MD), and in accordance with abnormal vestibular testing (calorics, vHIT). However, all remaining deficits were centrally compensated when patients were included in this study, which is reflected by similar normative results for posturography and SVV adjustment in both groups.

Skills and vestibular function were more preserved in p-PPPD, but the subjective symptom burden remained high and remarkable differences between p- and s-PPPD in terms of life quality as assessed by EQ5D-3L were lacking. However, overall quality of life was significantly reduced in both groups (VAS 58%) compared to healthy subject collectives, which is in line with earlier reports [43, 44]. The discrepancy between the high subjective burden in PPPD and the objective vestibular function is underlined by the lack of a significant correlation between DHI, VAP, and QoL scores and objective neurophysiological parameters (e.g., in vHIT and caloric testing). This is in line with earlier findings in patients with PPPD/somatoform vertigo that showed high fear of falling but not an objectively increased risk of falling in gait analyses [45].

BPPV (27%) followed by VM (24%) and acute unilateral vestibulopathy (16%) were the most important triggers for the development of s-PPPD. This result is partly in line with earlier studies. In their earlier study from 1995 on 154 patients with phobic postural vertigo, Huppert and co-workers also reported BPPV to be the most often associated organic vestibular disorder (44%) followed by acute unilateral vestibulopathy (32%) [6]. However, the diagnosis of VM was not established at that time [39]. A prospective study in organic vestibular diseases over one year also reported a significantly higher prevalence of psychiatric comorbidity, especially in patients with vestibular migraine, but not in those with BPPV [46]. Furthermore, another longitudinal study confirmed in a larger sample size that especially patients with VM are at risk of developing ongoing dizziness and psychological strain compared to other entities [47]. Thus, the high rate of preceding BPPV as a trigger for PPPD was unexpected. One may speculate that it is due to a snapshot at the time of the patients’ appearance in our outpatient unit, and that secondary functional dizziness in BPPV might fade away more rapidly when BPPV is successfully treated. The latter was shown previously [46] with elevated levels for anxiety and depression 6 weeks and 3 months after diagnosis and normalization afterwards. In addition, BPPV shows a higher prevalence in the general population [38] and might, therefore, be overrepresented in our patient cohort.

The phobic postural sway pattern in posturography analysis, which was the most common diagnostic finding (50%) in PPPD, is also noteworthy. This implies that most PPPD patients show typically poor performances during easy balance conditions (e.g., normal stance with eyes open and eyes closed) that improve with increasing difficulty or with additional cognitive tasks compared to healthy controls [26, 48]. Furthermore, posturography represents a helpful tool for longitudinal monitoring of a possible transition from an acute vestibular disorder to chronic functional dizziness, as demonstrated earlier by Brandt and co-workers [25]. Consequently, it appears advisable for all clinicians who do not have posturographic equipment available to clinically test stance performance also during more difficult and dual tasks [49].

The limitations of the current study are mainly its retrospective approach and a potential patient selection bias due to the referral of patients to a specialized tertiary center, which does not allow a transfer to the general population, especially in other countries. However, this study in a well-diagnosed PPPD cohort showed considerable differences in terms of demographic and clinical aspects between p- and s-PPPD patients, thereby underlining the need for careful syndrome classification and further prospective studies in clearly characterized p- and s-PPPD subgroups.
